# Identification of Bacterial Protein Interaction Partners Points to New Intracellular Functions of *Francisella tularensis* Glyceraldehyde-3-Phosphate Dehydrogenase

**DOI:** 10.3389/fmicb.2020.576618

**Published:** 2020-09-10

**Authors:** Monika Kopeckova, Ivona Pavkova, Marek Link, Pavel Rehulka, Jiri Stulik

**Affiliations:** Department of Molecular Pathology and Biology, Faculty of Military Health Sciences, University of Defence, Hradec Kralove, Czechia

**Keywords:** protein–protein interaction, multifunctional enzyme, *Francisella tularensis*, glyceraldehyde-3-phosphate dehydrogenase, SILAC

## Abstract

Glyceraldehyde-3-phosphate dehydrogenase (GAPDH) is well known for its involvement in numerous non-metabolic processes inside mammalian cells. Alternative functions of prokaryotic GAPDH are mainly deduced from its extracellular localization ability to bind to selected host proteins. Data on its participation in intracellular bacterial processes are scarce as there has been to date only one study dealing with this issue. We previously have reported several points of evidence that the GAPDH homolog of *Francisella tularensis* GapA might also exert additional non-enzymatic functions. Following on from our earlier observations we decided to identify GapA’s interacting partners within the bacterial proteome to explore its new roles at intracellular level. The quantitative proteomics approach based on stable isotope labeling of amino acids in cell culture (SILAC) in combination with affinity purification mass spectrometry enabled us to identify 18 proteins potentially interacting with GapA. Six of those interactions were further confirmed by alternative methods. Half of the identified proteins were involved in non-metabolic processes. Further analysis together with quantitative label-free comparative analysis of proteomes isolated from the wild-type strain strain with deleted *gapA* gene suggests that GapA is implicated in DNA repair processes. Absence of GapA promotes secretion of its most potent interaction partner the hypothetical protein with peptidase propeptide domain (PepSY) thereby indicating that it impacts on subcellular distribution of some proteins.

## Introduction

The glycolytic enzyme glyceraldehyde-3-phosphate dehydrogenase (GAPDH) is known for its ability to exhibit multiple unrelated functions within a single polypeptide chain (Pancholi and Chhatwal, 2003; Jeffery, 2009). In mammalian cells, it is involved in diverse cellular non-metabolic processes, including gene regulation, DNA repair, cytoskeletal dynamics, cell death, vesicular transport, and many others. These so-called moonlighting roles are associated with GAPDH’s unusual, non-predictable localization. Such multifunctionality also has been reported for bacterial GAPDH in a number of independent studies (Pancholi and Fischetti, 1992; Modun and Williams, 1999; Boël et al., 2005; Butland et al., 2005; Arifuzzaman et al., 2006; Jin et al., 2011; Ferreira et al., 2015). In some human pathogens, the surface-localized and/or -secreted GAPDH is able to bind to several host proteins (Kopeckova et al., 2020), thus contributing to its virulence. The mechanisms by which this protein is transported out of the cell remain obscure, however, because it lacks any known recognition motif for membrane and/or extracytosolic trafficking (Pancholi and Chhatwal, 2003). Data on the intracellular non-enzymatic functions of GAPDH in bacteria are also scant. The results of a transcriptomic analysis on *Streptococcus pyogenes* mutant strain displaying higher intracellular levels of GAPDH suggest it has a role in transcriptional modulation of virulence and metabolic genes (Boël et al., 2005). Several large-scale protein–protein interaction studies performed on *Escherichia coli* have revealed that bacterial GAPDH, similar to its eukaryotic orthologs or analogs, is able to interact with other intracellular proteins, including metabolic enzymes as well as proteins involved in transcription, protein synthesis, and DNA repair (Butland et al., 2005; Arifuzzaman et al., 2006; Ferreira et al., 2013, 2015).

The pathogenic bacterium *Francisella tularensis* is a facultative intracellular, Gram-negative coccobacillus that causes the zoonotic disease tularemia (McCrumb et al., 1957). The subspecies *tularensis* (type A) strains exhibit extreme virulence and a mortality rate potentially as high as 60% (Steiner et al., 2014). Due to the very low infectious doses and easy transmission by aerosol, this bacterium has been classified as a potential biological weapon (Ellis et al., 2002; Meibom and Charbit, 2010; Barel and Charbit, 2013). *F. tularensis* thus constitutes a serious threat for humanity, and this is further heightened by the absence of a licensed vaccine. A persistent lack of knowledge regarding the molecular mechanisms of tularemia’s pathogenesis, however, hampers the development of effective and safe prophylactics.

We previously have shown that the GAPDH homolog GapA contributes to the full virulence manifestation of the *F. tularensis* subsp. *holarctica* FSC200 strain (Pavkova et al., 2017). Furthermore, its demonstrated extracytosolic localization and ability to bind to several host serum proteins have indicated that GapA performs other functions beyond its primary enzymatic role in glycolysis. In the presented study, we aimed to identify bacterial protein interaction partners to elucidate the potential non-enzymatic roles of *F. tularensis* GapA protein inside the bacterial cell. We employed for this purpose a stable isotope labeling of amino acids in cell culture (SILAC) strategy (Ong et al., 2002) in combination with affinity purification followed by quantitative mass spectrometry (SILAC-AP-MS/MS). This approach enabled us to identify 18 potential GapA interaction partners implicated in various intracellular processes. Several of them were selected for further confirmatory and/or functional characterization. We additionally analyzed the impact of *gapA* gene deletion on the *F. tularensis* proteome by quantitative label-free proteomic comparative analysis. Our data indicate that in *F. tularensis* the GapA is likely engaged in DNA reparation processes and in compartmentalization of proteins.

## Materials and Methods

### Bacterial Strain, Plasmids, and Culture Conditions

The *F. tularensis* and *E. coli* strains and plasmids used in this study are listed in Supplementary Table S1. *Francisella* strains were cultured on McLeod agar enriched with bovine hemoglobin (Becton Dickinson, Cockeysville, MD, United States) and IsoVitaleX (Becton Dickinson) and in liquid Chamberlain’s chemically defined medium (CDM) (Chamberlain, 1965) at 37°C. If not further specified, the *E. coli* strains were grown on Luria–Bertani (LB, Sigma–Aldrich, Schnelldorf, Germany) agar and in liquid LB broth or Terrific Broth medium at 37°C. When necessary, the following antibiotics were used: chloramphenicol 2.5 μg/ml (*F. tularensis*) or 25 μg/ml (*E. coli*), polymyxin B 75 μg/ml (*F. tularensis*), carbenicillin 50 μg/ml (*F. tularensis*), kanamycin 50 μg/ml (*E. coli*) or 20 μg/ml (*F. tularensis*).

### Construction of *F. tularensis* Strain Expressing Strep-Tagged GapA Protein

The *gapA* gene (locus tag FTS_1117) was fused to the coding sequence of the Strep-tag (eight amino acids: WSHPQFEK) on its C-terminus using PCR amplification with specific primers (see Supplementary Table S2). The *gapA* gene (locus tag FTS_1117) was fused to the coding sequence of the Strep-tag (eight amino acids: WSHPQFEK) on its C-terminus using mutagenesis kit QuikChange II XL Site-Directed (Agilent, United States) with specific primers (see Supplementary Table S2). The final sequence (*gapA*-Strep) was introduced into the chromosome of *F. tularensis* FSC200 *gapA* deletion mutant strain (Δ*gapA*) prepared previously by Pavkova et al. (2017) by in-*cis* complementation strategy similar to in-frame deletion mutagenesis described below in section “Construction of In-Frame Deletion pepSY Mutant.” Briefly, the DNA construct encoding the *gapA*-Strep gene with introduced restriction sites *Xho*I and *Spe*I was generated by overlapping PCR amplification using the specific primers (Supplementary Table S2). The resulting DNA fragment was cloned into pCR4-TOPO vector (Supplementary Table S1) and verified by sequence analysis (ABI PRISM 3130xl, Applied Biosystems). Fragments from plasmids with verified inserts were cloned into suicide pDM4 plasmid (Supplementary Table S1), and then introduced into *E. coli S17-1*λ*pir* (Supplementary Table S1). The plasmids were mobilized into the Δ*gapA* by conjugation followed by sucrose selection resulting finally to the allelic exchange on the FSC200 chromosome.

### SILAC Metabolic Labeling, Cell Lysate Preparation, Affinity Purification

For metabolic labeling, the *F. tularensis* wild-type strain (wt) or strain expressing Strep-tagged GapA protein (FSC200/*gapA_*S) were cultivated in CDM containing the heavy labeled amino acids L-arginine hydrochloride [^13^C_6_^15^N_4_] and L-lysine hydrochloride [^13^C_6_^15^N_2_] (Sigma–Aldrich, Schnelldorf, Germany) in the same concentrations as in the light medium. After three generations, the incorporation levels were checked by mass spectrometry (MS) and the labeled bacteria were stored as frozen stocks at −150°C. For the experiment, the labeled bacterial stock was cultivated in heavy medium overnight at 37°C, 200 rpm. Non-labeled bacteria were grown in the light medium. The overnight cultures were diluted with fresh media to optical density at 600 nm (OD_600__*nm*_) = 0.1 and cultivated to OD_600__*nm*_ = 0.8 (approximately 3.2 × 10^9^ bacteria/ml, late exponential phase). Bacteria were then harvested by centrifugation and washed with phosphate-buffered saline (PBS, pH 7.4). At this point, the light and heavy bacteria were mixed in a 1:1 cell ratio. Three sets of biological replicates were prepared. Each set consisted of three samples. In two samples, the wt FSC200 was grown in light medium and the FSC200/*gapA_*S in heavy medium. For the third sample, the SILAC groups were swapped (FSC200/*gapA_*S was cultivated in light medium, wt FSC200 strain in heavy medium). After supplementation with protease inhibitor cocktail Complete EDTA-free (Roche Diagnostics, Mannheim, Germany) and benzonase (150 U/ml; Sigma–Aldrich), the bacterial cells were disrupted in a French press (Thermo IEC, Needham Heights, MA, United States) by two passages at 16,000 psi. Unbroken cells were removed by centrifugation at 6,000 × *g* for 40 min at 4°C. The clarified lysate supplemented with an appropriate amount of Avidin (11 U/mg; IBA Lifesciences, Göttingen, Germany) was applied on a column containing Strep-Tactin^®^ affinity resin and the GapA/S protein was purified using the Strep-tag^®^ purification system according to the manufacturer’s instructions (IBA Lifesciences).

### Preparation of the Whole-Cell Lysate, Crude Membrane Fraction, and Culture Filtrate Proteins

Bacteria were grown in CDM at 37°C until the late logarithmic phase (0.7–0.8 OD_600 *nm*_). To obtain whole-cell lysate, the bacteria were lysed in a French pressure cell and unbroken cells and cell debris were removed by centrifugation. The pellet with membrane proteins was obtained by ultracentrifugation of the whole-cell lysate, and then suspended in PBS as described previously (Pavkova et al., 2017). The supernatant after the ultracentrifugation was used as cytosolic fraction in Western blot analysis. For the preparation of culture filtrate proteins, the bacteria were removed by centrifugation and the culture medium was vacuum-filtered through membranes (0.2 μm pore; Merck Millipore, Billerica, MA, United States). The filtrate was then concentrated using Stirred Ultrafiltration Cell (8200, Merck Millipore) with 5 kDa cutoff membrane from regenerated cellulose (Merck Millipore) followed by diafiltration using Amicon Ultra 3K devices (Merck Millipore) to further concentrate the protein sample and exchange the medium for 40 mM Tris-HCl (pH 7.3). The protein content was determined using a bicinchoninic acid assay.

### Sample Preparation for Mass Spectrometry Analysis

The protein samples were adjusted with 25 mM ammonium bicarbonate with 10% (w/v) sodium deoxycholate monohydrate (DOC) to final concentration 1%. Samples were next reduced with 4 mM dithiothreitol (DTT) at 60°C for 45 min, at 700 rpm, then alkylated with 16 mM iodoacetamide at room temperature for 30 min in darkness. The unreacted iodoacetamide was quenched with an additional 4 mM DTT at room temperature for 30 min. The proteins were digested with trypsin (Promega, Madison, WI, United States) overnight at 37°C. Thereafter 1 M hydrochloric acid was added to stop the digestion and precipitate DOC. The suspension was then mixed with an equal volume of ethyl acetate, vortexed vigorously for 1 min, centrifuged at 14,000 × *g* for 5 min, and the upper organic layer was removed. The extraction was repeated three times to completely extract DOC. The aqueous phases were desalted on Empore^TM^ C18-SD (4 mm/1 ml) extraction cartridges (Sigma–Aldrich) and dried in a vacuum.

### Liquid Chromatography and Mass Spectrometry Analysis

The prepared bacterial peptide samples were dissolved in 20 μl 2% acetonitrile (ACN)/0.1% trifluoroacetic acid (TFA) and 1 μl was analyzed using the UltiMate 3000 high-performance liquid chromatography system (Dionex/Thermo Scientific, United States). This system with UV detection included a μ-Precolumn (300 μm × 5 mm, C18PepMap 5 μm 100 Å particles; Dionex) connected to the analytical NanoEase column (100 μm × 150 mm, Atlantis C18 3 μm 100 Å particles; Waters, Milford, MA, United States). Peptide separation was performed using the bilinear gradient of 3–44% ACN/0.1% TFA over 81 min under a flow rate of 360 nl/min and UV detection set to 215 nm. The data acquisition and chromatogram visualization were carried out in the Chromeleon software (v. 6.80, Dionex). The separation of peptides for nanoscale liquid chromatography-tandem mass spectrometry analysis was done using the UltiMate 3000 RSLC-nano high-performance liquid chromatography system (Dionex) with a trap column (75 μm × 20 mm) packed with 3 μm Acclaim PepMap100 C18 particles and a separation column (75 μm × 150 mm) packed with 2 μm Acclaim PepMap RSLC C18 particles. The samples were loaded onto the trap column with 2% ACN/0.05% TFA and peptide separation was performed with the dual linear gradient using 3–44% ACN in 0.1% formic acid over 89 min for all samples under the flow rate of 300 nl/min. Column temperature was set to 40°C. The separation was monitored with UV detection at 214 nm and directly coupled to mass spectrometry analysis with the Q Exactive system (Thermo Fisher Scientific, United States) in the positive mode with full MS scan (350–1650 m/z) at 70,000 full width at half maximum (FWHM), maximum filling time 100 ms and automatic gain control (AGC) target 1e6. The top 12 precursors in MS/MS at 17,500 FWHM with precursor charge 2+ or higher were selected for all samples with minimum threshold intensity of 5e4, maximum filling time 100 ms, and AGC target. Instrumental data acquisition was done using Xcalibur software v. 3.0.

### Data Processing and Protein Identification

#### SILAC Quantification

Peptides and proteins were identified by searching the raw files against the *F. tularensis* subsp. *holarctica* (wt FSC200) protein sequences database (1,423 entries, downloaded from UniProt) appended with common protein contaminants (245 sequences, downloaded from MaxQuant homepage) using the Mascot v2.3 search engine (Matrix Science, Boston, MA, United States) within the Proteome Discoverer v2.2 software (Thermo Scientific). The parameters were as follow: trypsin was used as the enzyme, two missed cleavages were allowed, carbamidomethylation of cysteine was set as a fixed modification, oxidation of methionine was selected as a variable modification, and the mass tolerance of the precursor and fragment ions was set to 20 ppm and 0.02 Da, respectively. Arginine (^13^C_6_, ^15^N_4_) and lysine (^13^C_6_, ^15^N_2_) were set as labels in the heavy channel for peptide quantitation. A false discovery rate of 0.01, estimated by target-decoy approach, was used for accepting the identifications on the peptide-to-spectrum match and protein levels. The SILAC-based approach was used to reveal the candidate interacting proteins of GapA. These are expected to be detected in a single SILAC quantitation channel, contrary to background proteins, resulting in extreme abundance ratio values. Therefore, the low abundance resampling algorithm of the Proteome Discoverer was used to impute missing values of single-channel quantitative data on the peptide level. The GapA interacting proteins were found as significantly up-regulated proteins using a global mean rank test (Klammer et al., 2014) (R package MeanRankTest^1^) with parametric FDR level set to 0.05. Only the *Francisella* proteins quantified in all four replicates were allowed for testing. Data visualization was done in Perseus v1.6.2.3 software (Tyanova et al., 2016).

#### Label-Free Quantification

Proteome Discoverer software (Thermo Fisher Scientific, v. 2.4.0.305) was used for identification of MS/MS spectra and label-free quantification of the measured data. The raw files from the QExactive mass spectrometer were processed within the processing workflow containing spectrum files RC, spectrum selector, non-fragment filter, top N peaks filter, precursor detector, SequestHT search engine, and target decoy PSM validator. The parameters for SequestHT database searching were: protein database – UniProt Francisella tularensis subsp. holarctica FSC200 reference proteome UP000006302 (11 May 2018); enzyme – trypsin; maximum missed cleavage sites – 1; min. peptide length – 7; precursor mass tolerance – 15 ppm; fragment mass tolerance – 0.02 Da; weight of b- and y-ions – 1; static modifications – carbamidomethyl/ +57.021 (C); dynamic modifications – oxidation/ +15.995 Da (M); dynamic modifications (protein terminus) – acetyl/ +42.011 Da (N-terminus), Met-loss/-131.040 Da (M), Met-loss + acetyl/-89.030 Da (M); and dynamic modifications (peptide terminus) – Gln- > pyro-Glu/-17.027 Da (Q). The search results obtained in the msf file were further processed in the consensus workflow containing PSM Grouper, Peptide Validator, Protein and Peptide Filter, Protein Scorer, Protein FDR Validator, Protein Grouping, Protein in Peptide Annotation Nodes, Protein Marker, Modification Sites, Feature Mapper, and Precursor Ions Quantifier. Only proteins identified with at least two peptides at FDR level 0.01 with *p*-value less than 0.01 of the quantitation change (computed from peptide abundances in Proteome Discoverer software) greater than 1.5 were considered. The heat maps were visualized in the Proteome Discoverer graphic tools for Venn diagrams reports.

The raw MS data as well as the processed identification results have been deposited to the ProteomeXchange Consortium via the PRIDE partner repository with the dataset identifier PXD019739.

### Expression and Purification of Recombinant Proteins Derived From *F. tularensis*

The genes encoding selected *F. tularensis* proteins were amplified by PCR with specific primers (Supplementary Table S2), and the gel-purified PCR products (Qiagen) were inserted into pET28b using either the *Nco*I and *Xho*I restriction sites (*pepSY, rho, pepB*, gene of YCII-related domain protein) or *Hin*dIII and *Xho*I restriction sites (*trxA*). The resulting plasmids encoded the aforementioned proteins with a C-terminal histidine tag. The final plasmid constructs were verified by direct DNA sequencing. For protein expression, the plasmids were transformed into *E. coli* NiCo21 expression strains (New England BioLabs, Ipswich, AM, United States). The cells were grown in Terrific Broth medium at 28°C overnight. Bacteria were lysed in a French pressure cell and the lysates were applied to a TALON^®^ Metal Affinity Resin purification system (Clontech, Mountain View, CA, United States) for purification of His-tagged proteins. Finally, the elution buffer was changed to T_100_N_150_ (100 mM Tris-HCl, 150 mM NaCl, [pH 7.6]) using an Amicon^®^ Ultra-15 centrifugal filter (Sigma–Aldrich). Eluted proteins were verified by SDS-PAGE followed by Coomassie staining (data not shown), and the protein concentrations were determined using Coomassie Plus Bradford assay reagent (Thermo Fisher).

### Solid-Phase Ligand-Binding Assay

This assay was used to confirm the ability of GapA to bind to selected *F. tularensis* proteins and was conducted as described previously (Pavkova et al., 2017). Briefly, the purified recombinant proteins were coated onto a 96-well high binding microtiter plate overnight and then incubated with recombinant GapA (0.25–2.5 μg/ml). The amount of GapA bound to the proteins was determined spectrophotometrically (450 nm) in an enzyme-linked immunosorbent assay using anti-GapA antibody (Apronex, Vestec, Czechia).

### Bacterial Two-Hybrid System

To confirm the interaction of GapA with UvrA, we performed bacterial two-hybrid assay using the BACTH (bacterial adenylate cyclase two-hybrid) System kit (Euromedex, Strasbourg, France). The *uvrA* and *gapA* genes were amplified from *F. tularensis* FSC200 genomic DNA, then inserted into the pKT25 and pUT18C using *Kpn*I and *Bam*HI restriction sites. The resulting plasmids were transformed into the BTH101 cells. Further protein–protein interaction analysis was performed according to the manufacturer’s protocols.

### UV Tolerance Assay

The effect of UV on the survival of selected *F. tularensis* strains was evaluated as described by Karki and Ham (2016), albeit with minor modifications. The overnight bacterial cultures were resuspended in PBS to a density of OD_600__*nm*_ = 1.0 (approximately 4 × 10^9^ bacteria/ml) and 10-fold serial dilutions of this bacterial suspension were plated onto McLeod agar. The inoculated plates (with open lids) were positioned directly below the UVLMS-38EL Series 3UVTM Lamp 8W (UVP, Cambridge, United Kingdom), at a distance of 37 cm, giving an irradiance of approximately 210 μW/cm^2^ at 254 nm. The bacteria were exposed to increasing applied doses of 254 nm UV light, using exposure times 0 s, 15 s, 30 s 60 s and 150 s, with dose calculated as irradiance (W/cm^2^) × exposure time (s). The plates were then incubated at 37°C in 5% CO_2_ for 5 days for CFU enumeration of the surviving bacteria.

### H_2_O_2_ Susceptibility Assay

The bacteria were first cultured in CDM overnight at 37°C. The next morning, the bacterial culture was centrifuged and resuspended in fresh CDM to OD_600 *nm*_ = 0.1 (approximately 4 × 10^8^ bacteria/ml). The hydrogen peroxide was added at final concentration 1 mM and the bacteria were cultivated at 37°C, 200 rpm. After 0, 2.5, and 5 h of incubation, bacterial samples were collected and viable bacteria determined by plating 10-fold serial dilutions. The plates were incubated for 5 days at 37°C in 5% CO_2_ before enumeration of CFU.

### Construction of In-Frame Deletion pepSY Mutant

The DNA construct encoding in-frame deletion for the *pepSY* gene with introduced restriction sites *Xho*I and *Spe*I (locus tag FTS_1731) was generated by overlapping PCR amplification using the specific primers (Supplementary Table S2). The resulting DNA fragment was cloned into pCR4-TOPO vector (Invitrogen, Carlsbad, CA, United States) and verified by sequence analysis (ABI PRISM 3130xl, Applied Biosystems, Foster City, CA, United States). Fragments from plasmids with verified inserts were cloned into the suicide pDM4 vector using the same restriction sites (Supplementary Table S1) and then introduced into *E. coli* S17-1γpir. The plasmids were mobilized into the *F. tularensis* FSC200 strain by conjugation followed by sucrose selection resulting finally to the allelic exchange on the FSC200 chromosome. Preparation of plasmid DNA, restriction enzyme digests, ligations, and transformations into *E. coli* all were performed essentially as previously described (Sambrook and Russell, 2001).

### Intracellular Replication and Invasion Assays

To generate bone marrow macrophages (BMMs), bone marrow cells were collected from dissected femurs of female BALB/c mice 6–10 weeks old and differentiated into macrophages in Dulbecco’s Modified Eagle Medium (DMEM, Invitrogen) supplemented with 10% fetal bovine serum and 20% L929-conditioned medium for 6–7 days. The differentiated BMMs were seeded at concentration 5 × 10^5^ cells/well in 24-well plates and infected the next day with wt FSC200 at multiplicity of infection 50:1 (bacteria/cell). To synchronize infection, the infected cells were centrifuged at 400 × *g* for 5 min and incubated at 37°C for 30 min. The extracellular bacteria were then removed by gentamicin treatment (5 μg/ml) for 30 min. For the proliferation assay, the infected BMMs were lysed at selected time points with 0.1% DOC. To determine numbers of intracellular bacteria, the lysates were serially diluted and plated onto McLeod agar.

### Animal Studies

For survival studies, groups of at least five female BALB/c mice 6–8 weeks’ old were infected subcutaneously with the Δ*pepSY* mutant strain (using doses of 10^2^ CFU/mouse) or the wt FSC200 strain. Control groups of mice were inoculated with sterile saline only. Mice were housed in micro-isolator cages and provided sterilized water and food *ad libitum*.

### Ethics Statement

This study was carried out in accordance with the recommendations of the guidelines of the Animal Care and Use Ethical Committee of the Faculty of Military Health Sciences, University of Defence, Czechia. The protocol was approved by the Ethical Committee of the Faculty of Military Health Sciences, University of Defence, Czechia.

### Western Blotting

Protein samples (2 μg of whole-cell lysate or cytosol, 40 μg of crude membrane fraction, 5 μg of culture filtrate proteins for each strain) were separated on 12% acryl-amide gels and transferred to polyvinylidene difluoride membranes (Bio-Rad Laboratories, Hercules, CA, United States). Membranes were blocked with 5% w/v skim milk powder, 0.1% v/v Tween in Tris-buffered saline and incubated in the same buffer with polyclonal rabbit anti-GapA antibody 1:1000 and swine anti-rabbit IgG/HRP (Dako, Glostrup, Denmark) 1:1000 as secondary antibody. Chemiluminescence was detected using a BM Chemiluminescence Blotting Substrate (POD) according to the manufacturer’s instructions (Roche Diagnostics). The blots obtained from biological duplicates were captured and analyzed using iBright^TM^ FL1000 Imaging System. Local background corrected density was used for band quantitation.

### Statistical Analysis

The assays were performed in triplicate for each strain and time. Each experiment was independently repeated at least three times unless otherwise stated. Values are expressed as mean ± standard deviation (SD) and analyzed for significance using Student’s two-tailed *t*-test. Differences were considered statistically significant at *p* < 0.05.

## Results

### Identification of GapA Protein Interaction Partners Using SILAC-AP-MS/MS Strategy

**FIGURE 1:**
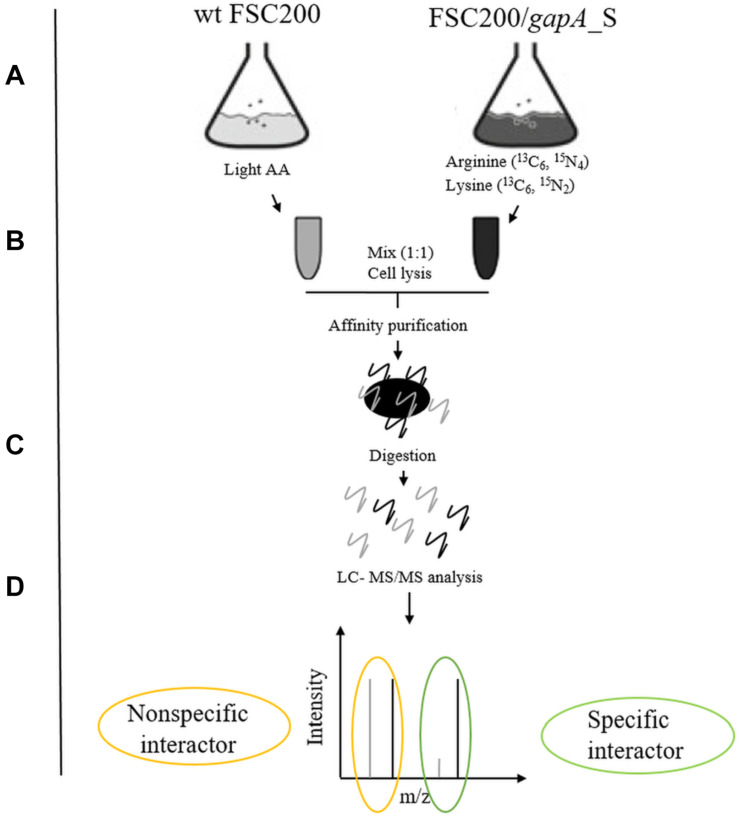
Schematic overview of SILAC-AP-MS/MS strategy. **(A)** The bacterial strains wt FSC200 and FSC200/*gapA_*S were grown in CDM with light or heavy amino acids (arginine ^13^C_6_, ^15^N_4_; lysine ^13^C_6_, ^15^N_2_). **(B)** Bacterial cultures (OD_600 *nm*_ = 0.8) were mixed in 1:1 cell ratio and lysed by French press. **(C)** GapA/S together with interacting partners was purified using Strep-tag^®^ purification system and digested with trypsin. **(D)** Tryptic peptides were analyzed by LC-MS/MS.

We used a quantitative proteomic approach based on SILAC metabolic labeling in combination with affinity purification and mass spectrometry to screen for bacterial proteins that bind specifically to the GapA of *F. tularensis* (Figure 1). The interconnection of these techniques enables confident discrimination between specific and non-specific interactions (Gingras et al., 2007; Auweter et al., 2011). For this purpose, we first engineered *F. tularensis* FSC200 to express GapA-Streptag fusion protein. The wt FSC200 and the mutant strain FSC200/GapA/S were grown in CDM containing arginine and lysine labeled with regular abundance or heavy isotopes, respectively. The two bacterial cultures were mixed together (1:1 cell ratio) and lysed. The GapA together with its binding partners was purified using StrepTactin. The effector complexes were treated with trypsin followed by LC-MS/MS analysis. Proteins bonding specifically to the Gap/S were significantly enriched in heavy labeled amino acids, resulting in high heavy:light SILAC ratios, whereas proteins that interacted non-specifically with the Strep-Tactin showed equal peak intensities for heavy and light forms.

Based on the criteria described in the section “Materials and Methods,” we were able to detect 18 potential binding partners (Table 1). The functional classification revealed that the largest group of the identified proteins (nine proteins) were involved in metabolic processes and might be related to the enzymatic function of GapA. The identified phosphoglycerate kinase forms in complex with GAPDH a functional ATP-generating unit that provides ATP production for glycolysis and might thus be considered as a method validation indicator. Another five proteins are engaged in genetic information processing, like transcription, translation, DNA repair, or protein folding. The remaining four proteins are only poorly characterized, but the presence of a functional domain in three of them indicates their putative role also in transcription processes.

**TABLE 1:** Candidate GapA interaction partners identified by SILAC-AP-MS/MS strategy.

Accession number (NCBI)	FTS locus tag	Protein name (alternative protein name/gene name)	Function*	Functional category
AFT92904	1118	Phosphoglycerate kinase	Carbohydrate metabolism: glycolysis/gluconeogenesis	**Metabolism**
AFT92415	0489	Glycogen phosphorylase	Carbohydrate metabolism: starch and sucrose metabolism	
AFT93363	1744	Citrate synthase	Carbohydrate metabolism: citrate cycle	
AFT92872	1077	Cytosol aminopeptidase (PepB)	Amino acid metabolism: glutathione	
AFT92409	0483	Shikimate 5-dehydrogenase	Amino acid metabolism: phenylalanine, tyrosine, tryptophan	
AFT92896	1110	3-Oxoacyl-(acyl-carrier-protein) reductase	Lipid metabolism: fatty acid biosynthesis	
AFT93113	1407	Phosphosugar isomerase (arabinose-5-phosphate isomerase, KdsD)	Glycan biosynthesis and metabolism, lipopolysaccharide biosynthesis	
AFT92505	0605	dTDP-glucose 4,6-dehydratase (rfbB, wbtM)	Secondary metabolites biosynthesis: metabolism of terpenoids and polyketides	
AFT92847	1040	Fumarylacetoacetate hydrolase family protein	Secondary metabolites biosynthesis, transport and catabolism	
AFT93130	1439	UvrABC system protein A (UvrA)	Replication and repair: nucleotide excision repair	**Genetic information processing**
AFT92508	0609	Transcription termination factor Rho (Rho)	Transcription: mRNA degradation factor	
AFT92389	0458	Cold shock protein (CspC)	Transcription	
AFT92201	0206	Valyl-tRNA synthetase	Translation	
AFT92509	0610	Thioredoxin	Chaperone and folding catalysts: protein disulfide isomerase	
AFT92431	0513	BolA family protein	Uncharacterized (? DNA binding regulator)	**Unknown**
AFT92893	1107	Hypothetical protein (YCII-related domain protein)	Uncharacterized (? YCII-related transcription regulation)	
AFT92074	0032	Hypothetical protein (ATPase family protein)	Uncharacterized (? transcription regulation)	
AFT93352	1731	Hypothetical protein (peptidase propeptide and YPEB domain protein, PepSY)	Uncharacterized	

*Function of protein as stated in NCBI or UniProt databases or predicted by KEGG pathway database.

### Confirmation of Selected Interactions

**FIGURE 2:**
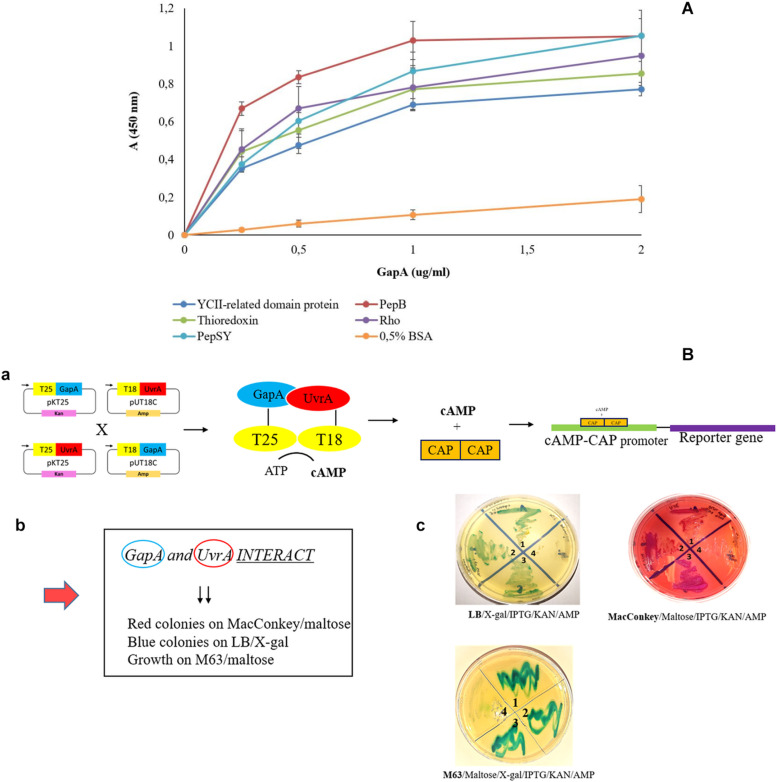
Validation of *F. tularensis* GapA binding ability to selected bacterial proteins. **(A)** Solid-phase binding assay of PepSY, Rho, thioredoxin, PepB, YCII-related domain protein, and BSA (negative control) coated on 96-well microtiter plate and reacted with different concentrations of purified GapA. Data are presented as means ± SD from three independent experiments. **(B)** Validation of GapA and UvrA interaction using the BACTH system. **(a)** Principle of the method: *GapA* and *uvrA* genes were cloned into the plasmids pKT25 and pUT18C and fused with T25 and T18 fragments. During the interaction of GapA with UvrA, complementation occurs between T25 and T18 fragments with subsequent cAMP synthesis. The complex of cAMP/CAP is a pleiotropic regulator of gene expression (*E. coli*) that initiates expression of reporter genes (*lac*, *mal* operon). **(b)** Manifestation of positive interaction. **(c)** BTH101 cells were co-transformed with the following plasmid combinations: *(1)* pKT25-*gapA* × pUT18C-*uvrA*, *(2)* pUT18C-*gapA* × pKT25-*uvrA*, *(3)* positive control plasmids pKT25 zip × pUT18C zip, and *(4)* negative control plasmids pkT25 empty + pUT18C empty. Co-transformed BTH101 cells with a plasmid combination were grown on selective agar plates (LB/X-gal/IPTG/KAN/AMP or MacConkey/Maltose/IPTG/KAN/AMP or M63/Maltose/X-gal/IPTG/KAN/AMP). The bacteria grown on LB agar plates were passaged on MacConkey agar plates and vice versa. We observed the results to be the same (data not shown).

To validate our proteomic screen, we further aimed to confirm the GapA interaction with the seven most potent hits based on significance in the global mean rank test. Using the solid-phase ligand-binding assay, we were able to confirm the binding ability of purified recombinant GapA with the protein candidates PepSY, Rho, thioredoxin, PepB, and YCII-related domain protein *in vitro* (Figure 2A). Due to problems with purification of recombinant UvrA, this interaction was successfully confirmed using the bacterial two-hybrid system (Figure 2B).

### Sensitivity of GapA-Deficient Cells to DNA Damaging Conditions

**FIGURE 3:**
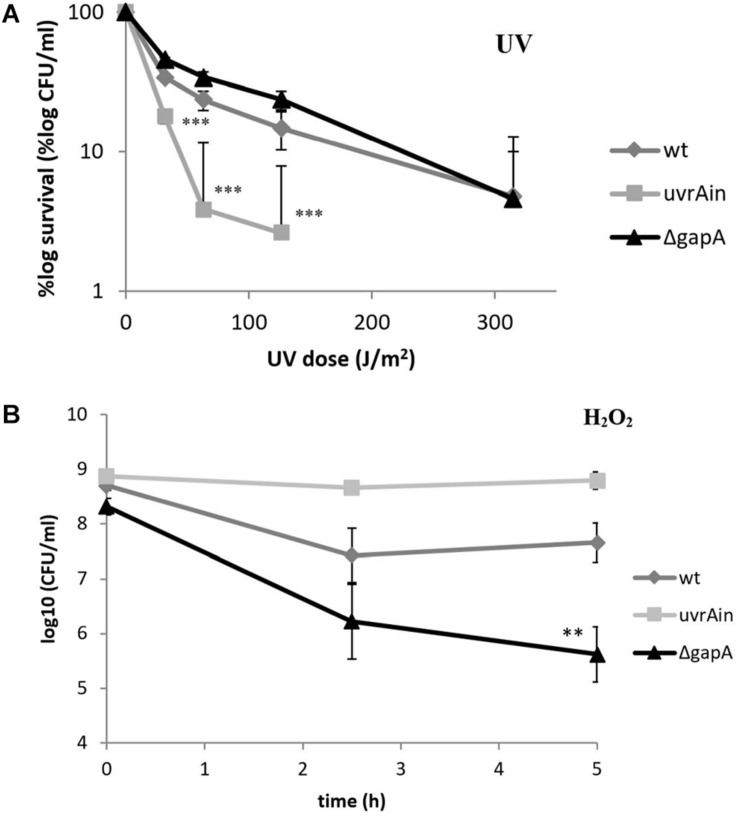
Sensitivity of *F. tularensis* Δ*gapA*, wtFSC200, and *uvrA*in to UV irradiation **(A)** and to H_2_O_2_ **(B)**. For UV tolerance assay **(A)**, 10-fold serially diluted bacteria plated on McLeod agar were irradiated for various lengths of time under a 254 nm germicidal lamp. After UV irradiation the bacteria were cultivated at 37°C and the number of survivors was calculated by plate counts. For the *uvrA*in, no viable colonies could be detected when irradiated for 150 s. For the H_2_O_2_ susceptibility assay **(B)**, bacteria in CDM were exposed to 1 mM H_2_O_2_ for 2.5 and 5 h. The viable bacteria were determined by plating 10-fold serial dilutions. The values shown in both graphs represent means of triplicates ± SD. Three independent experiments were performed. Asterisks indicate statistically significant differences according to the Student’s two-tailed *t*-test: **P < 0.01; ***P < 0.001 (comparing mutant strain with the wt strain).

The UvrA protein identified in our study as a potential GapA interacting partner forms together with UvrB and UvrC proteins a multienzyme complex responsible for nucleotide excision repair (NER) in DNA damaged primarily by ultraviolet (UV) radiation (Sancar and Reardon, 2004). We thus decided to examine the sensitivity of bacteria with deleted *gapA* gene to UV irradiation. As seen in Figure 3A, however, the survival rates of the Δ*gapA* mutant were almost comparable to those of wt FSC200. In contrast, the mutant strain with disrupted *uvrA* gene (*uvrA* insertion mutant – *uvrA*in), published previously in use as a positive control (Pavkova et al., 2017), was far more sensitive to UV killing. None of the three strains showed any difference in their susceptibility to alkylating agent methyl methanesulfonate (data not shown). As DNA damage caused by alkylating agents is predominantly repaired by the base excision repair pathway, this indicates that neither the UvrA nor GapA are essential for this DNA repair process.

In contrast, the Δ*gapA* was observed to be significantly more sensitive upon exposure to H_2_O_2_, a highly reactive molecule inducing oxidative DNA lesions. The viability of Δ*gapA* treated with 1 mM H_2_O_2_ gradually decreased by about 2.7 log_10_ during the 5 h of cultivation. Meanwhile, the viability of the wt strain dropped only during the first 2.5 h to by about 1.3 log_10_ and then ceased to decline (Figure 3B). Notably, the viability of *uvrA*in remained nearly undisturbed. The concentration used had previously been shown not to affect the viability of virulent strains wt FSC200 and SchuS4 (Lindgren et al., 2007).

### The PepSY Protein Is Not Essential for *F. tularensis* Virulence and Does Not Affect the GapA Extracellular Localization

**FIGURE 4:**
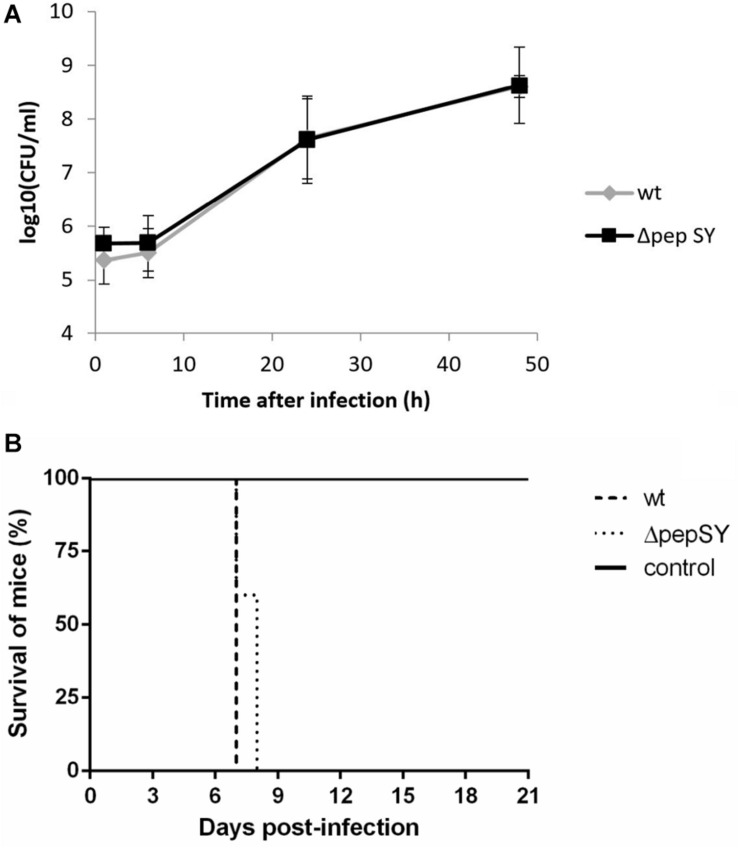
*In vitro* proliferation of Δ*pepSY* and wt FSC200 in BMMs **(A)**. The cells were infected at multiplicity of infection 50:1 with the indicated strains. BMMs were harvested at 1, 6, 24, and 48 h. The data shown are means ± SD of triplicate samples and the results shown are representatives of three independent experiments. Survival of mice infected with ΔpepSY mutant **(B)**. Groups of five BALB/c mice were infected subcutaneously with the wt and ΔpepSY mutant using a dose of 10^2^ CFU/mouse, the control group was inoculated with sterile saline.

The protein displaying the highest significance score in the global mean rank test of the SILAC-AP-MS/MS analysis was the PepSY domain-containing protein (hypothetical protein FTS_1731, PepSY). According to UniProtKB, the protein contains a signal sequence and two PepSY domains^2^. Although this domain is believed to have a protease inhibitory function, the role of PepSY-containing proteins is not clear to date. We wanted to test whether this protein might play a role in *F. tularensis* virulence, and therefore we introduced the *pepSY* in-frame deletion mutant in strain wt FSC200 (Δ*pepSY*). The Δ*pepSY* mutant was shown to enter and replicate in BMMs to the same extent as did the wt strain (Figure 4A). In addition, all mice infected subcutaneously with the mutant strain at doses 10^2^ CFU died within 7–8 days, as did mice infected with the equal dose of wt strain (Figure 4B). Based on our observations, deletion of the gene encoding the PepSY protein did not result in any obvious changes in *F. tularensis* phenotype and thus that protein is not essential for the virulence of this pathogen.

**FIGURE 5:**
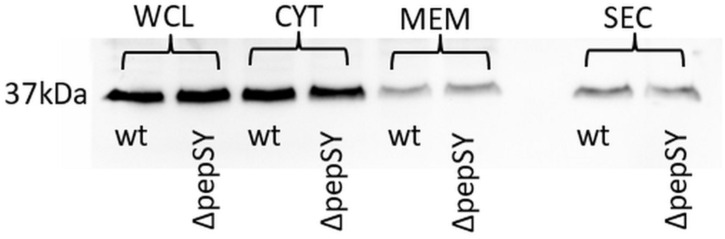
Representative immunodetection of GapA protein (Mw 37 kDa) in whole-cell lysate (WCL), cytosol (CYT), crude membrane fraction (MEM), and culture filtrate protein (SEC) isolated from *F. tularensis* FSC200 wt or ΔpepSY mutant strain. Protein samples (2 μg of WCL and CYT, 40 μg of MEM, 5 μg of SEC for each strain) were separated on 12% SDS-PAGE. Western blots were probed with polyclonal rabbit anti-GapA antibody (Apronex), imaged and analyzed using the iBright Imaging System (Invitrogen).

In a previously published study, we were able to demonstrate extracellular localization of *F. tularensis* GapA. The GapA sequence, however, lacks a signal sequence or other known sorting motif for extracytosolic trafficking, so the mechanism of its export to the exterior of the cell is unclear. As a signal sequence was predicted in the PepSY, we hypothesized that this protein could assist GapA through the bacterial membrane. To follow this hypothesis, we compared the GapA abundance in the Δ*pepSY* and wt strains. The whole-cell lysates, cytosolic proteins, crude membrane fraction, and culture filtrate proteins were separated on SDS-PAGE, which was followed by Western blot and immunodetection of GapA with polyclonal antibody. The Western blot analysis did not reveal any significant changes in GapA distribution across all the analyzed fractions between the wt and Δ*pepSY* strain (Figure 5). It might thus be suggested that the PepSY protein has no impact on GapA localization.

### Impact of *gapA* Gene Deletion on *F. tularensis* Proteome

**FIGURE 6:**
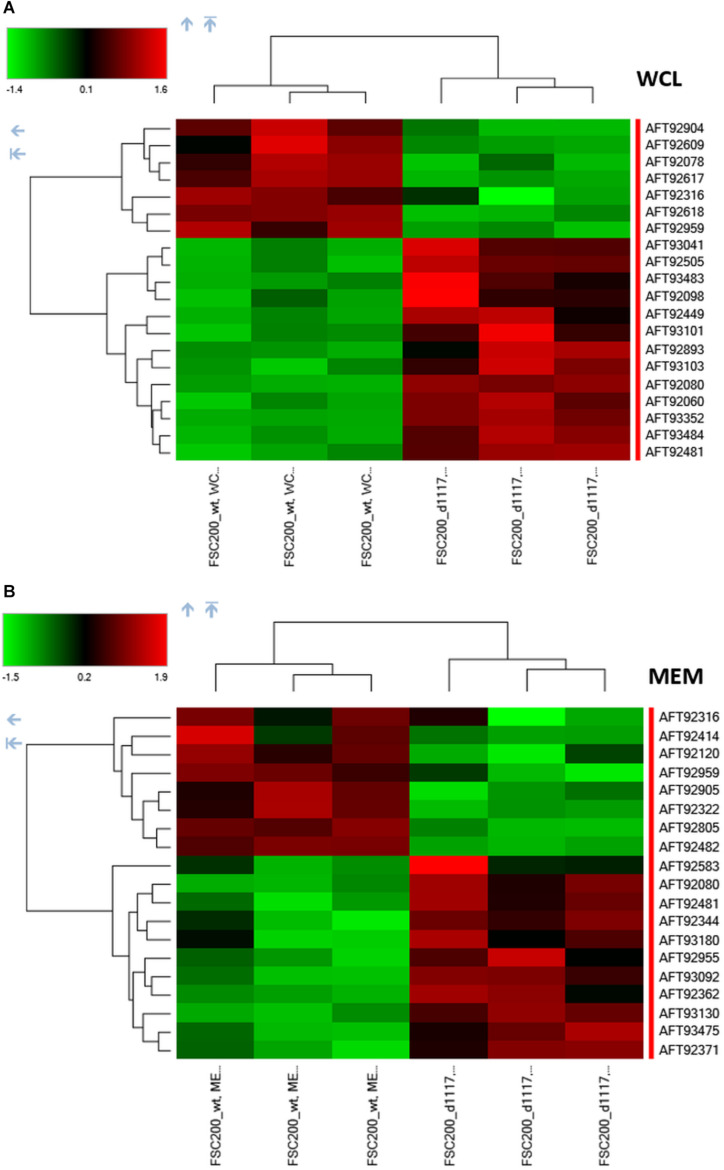
Biclustering and heat maps of proteins differentially expressed and identified from label-free MS analysis of whole-cell lysates **(A)** and crude membrane fractions **(B)** of wt FSC200 and Δ*gapA* strains.

In order to reveal the broader spectrum of GapA functions, we also decided to analyze the impact of *gapA* gene deletion on *F. tularensis* proteome. For this purpose, we performed a comparative nanoscale liquid chromatography-tandem mass spectrometry analysis of whole-cell lysates, crude membrane fractions, and culture filtrate proteins obtained from the FSC200 wt and Δ*gapA* mutant strains. We were able to identify in total 1,092 proteins in whole-cell lysates, 985 proteins in crude membrane fractions, and 748 proteins in culture filtrates. The label-free quantification approach enabled us to select 38 proteins whose amounts significantly differed between the two tested strains (Table 2). In whole-cell lysates, 13 proteins were significantly more and seven proteins significantly less abundant in Δ*gapA* compared to wt. To increase the depth of our proteome analysis we have also analyzed the crude membrane fraction. This sample enrichment enabled in the identification of additional 16 differently abundant proteins – 10 of them were significantly increased and 6 decreased in the mutant strain. Otherwise, it might also be assumed that the distribution of some proteins had changed as a consequence of *gapA* deletion. The biclustering analysis and heat maps obtained by comparing the differentially expressed proteins in these two fractions revealed that the protein profile of the Δ*gapA* was different from that of the wt FSC200 strain (Figure 6). The analysis of culture filtrate proteins revealed an extremely increased abundance of PepSY in Δ*gapA*, indicating that PepSY secretion might be related to the absence of GapA in Δ*gapA.* These findings taken together suggest that GapA really is able to affect the compartmentalization of other proteins.

**TABLE 2:** Proteins with significantly altered expression in Δ*gapA* compared to the FSC200 wt strain detected by label-free quantitative shotgun approach.

Accession number (NCBI)	Protein name (gene name)	FTS locus tag	Abundance ratio ΔgapA/wt	Adjusted *p*-value	Functional category
**Whole-cell lysate**					
AFT93352	*Hypothetical protein – PepSY*	1731	4.814	1.35E-15	Unknown (peptidase propeptide and YPEB domain protein). 2 PepSY domains
AFT92080	Chorismate mutase	0041	3.003	4.25E-11	Chorismate metabolic process
AFT92481	Sugar porter (SP) family protein	0580	2.216	9.75E-06	Transmembrane transporter activity
AFT93484	Hypothetical protein	1908	2.137	3.08E-05	Unknown
AFT92449	Uracil-DNA glycosylase (*ung*)	0533	2.107	4.47E-05	DNA replication and repair
AFT93483	Hypothetical protein	1907	2.091	5.19E-05	Uncharacterized
AFT92893	*Hypothetical protein*	1107	2.002	2.02E-04	Unknown
AFT93101	Haloacid dehalogenase-like hydrolase	1395	1.959	3.34E-04	Poorly characterized
AFT93041	Alanine racemase	1305	1.953	3.55E-04	D-Alanine metabolism
AFT93103	Glycosyl transferase family protein	1397	1.871	1.17E-03	Uncharacterized
AFT92060	Inhibitor of RecA (*recX*)	0012	1.782	4.11E-03	DNA repair and recombination
AFT92505	*dTDP-D-glucose 4.6-dehydratase* (*rfbB. wbtM*)	0605	1.777	4.27E-03	Nucleotide-sugar metabolic process
AFT92098	Riboflavin synthase beta-chain (*ribH*)	0067	1.750	5.99E-03	Riboflavin metabolism
AFT92316	LemA-like protein (lemA)	0357	0.548	3.83E-03	Poorly characterized
AFT92078	Hypothetical protein	0037	0.497	2.82E-04	Unknown
AFT92959	Hypothetical protein	1192	0.467	4.65E-05	Unknown
AFT92904	*Phosphoglycerate kinase (pgk)*	1118	0.452	1.92E-05	Carbohydrate metabolism – glycolysis/gluconeogenesis
AFT92618	Excinuclease ABC subunit B (*uvrB*)	0745	0.371	1.22E-08	DNA replication and repair
AFT92617	Exodeoxyribonuclease I (*sbcB*)	0744	0.336	1.50E-10	DNA replication and repair
AFT92609	Lactoylglutathione lyase (*gloA*)	0732	0.283	2.66E-14	Carbohydrate metabolism: pyruvate metabolism
**Crude membrane fraction**					
AFT93092	Recombination protein RecR (*recR*)	1381	3.542	3.35E-06	DNA repair and recombination
AFT92371	Riboflavin biosynthesis protein RibF (*ribF*)	0427	3.111	3.99E-09	Riboflavin metabolism
AFT92583	Hypothetical protein	0703	2.261	1.59E-04	Unknown
AFT93180	2-Dehydro-3-deoxyphosphooctonate aldolase (*kdsA*)	1499	2.173	1.79E-03	Glycan biosynthesis and metabolism: lipopolysaccharide biosynthesis
AFT92481	Sugar porter (SP) family protein	0580	2.113	8.17E-03	Transmembrane transporter activity
AFT93130	*Excinuclease ABC. subunit A (uvrA)*	1439	2.058	6.63E-04	DNA replication and repair
AFT92080	Chorismate mutase	0041	2.009	1.22E-03	Chorismate metabolic process
AFT92955	Leucyl-tRNA synthetase (*leuS*)	1186	2.004	1.22E-03	Translation
AFT92362	DNA topoisomerase I (*topA*)	0417	1.967	2.88E-03	DNA repair and recombination
AFT93475	Putative ABC transporter ATP-binding protein	1893	1.947	2.15E-03	Transfer RNA biogenesis
AFT92344	glucose kinase (*glk*)	0395	1.935	2.41E-03	Carbohydrate metabolism – glycolysis/gluconeogenesis
AFT92120	IglA protein (*iglA1*)	0103	0.501	4.44E-03	Type VI secretion system component
AFT92316	LemA-like protein (lemA)	0357	0.498	3.91E-03	Poorly characterized
AFT92322	Hypothetical protein	0366	0.483	9.46E-03	Unknown
AFT92482	Hypothetical protein	0581	0.464	1.88E-03	Unknown
AFT92805	Oxidative stress transcriptional regulator (*oxyR*)	0989	0.450	4.64E-04	Transcription factor
AFT92959	Hypothetical protein	1192	0.399	1.79E-05	Unknown
AFT92414	Glycogen synthase (*glgA*)	0488	0.344	1.55E-05	Carbohydrate metabolism – starch and sucrose metabolism
AFT92905	Pyruvate kinase (*pyk*)	1119	0.322	1.39E-08	Carbohydrate metabolism – glycolysis/gluconeogenesis
**Culture filtrate proteins**					
AFT93352	*Hypothetical protein – PepSY*	1731	22.438	6.59E-03	Unknown (peptidase propeptide and YPEB domain protein). 2 PepSY domains
AFT93070	Hypothetical protein	1352	0.018	2.56E-07	Chorismate metabolic process (Chorismate mutase type II family protein)

*Protein names highlighted in italic were also identified as potential GapA interaction partners.*

Based on the functional classification, deletion of the *gapA* gene resulted in differentially expressed proteins involved in metabolic (12 proteins) and genetic information processing (11 proteins). Another 13 proteins were only poorly characterized and their function remains so far unknown. As expected, proteins of the carbohydrate metabolic pathway were the most under-represented in the proteome of the Δ*gapA* strain. The changed levels of several enzymes engaged in other metabolic processes might indicate the effort of *F. tularensis* to compensate the disturbed glycolytic pathway. Interestingly, most of the proteins from the functional category “genetic information processing” are engaged in distinct DNA reparation processes (UvrA, UvrB, uracil-DNA glycosylase, exodeoxyribonuclease I, RecR, RecX). Four proteins significantly increased in response to *gapA* deletion were also identified as potential interaction partners: UvrA, hypothetical YCII-related protein, PepSY, and dTDP-glucose 4,6-dehydratase. This finding supports the assumption that these proteins interact with GapA.

## Discussion

Although moonlighting of the eukaryotic GAPDH is a well-known phenomenon reported in a number of published studies, current knowledge about the non-enzymatic roles of GAPDH in prokaryotes remains limited. Several studies have been able to demonstrate the involvement of this protein in host cell manipulation (Terao et al., 2006; Alvarez-Dominguez et al., 2008; Boradia et al., 2014), but its potential role in other non-metabolic processes at the intracellular level remains nearly unexplored. Because GAPDH has no functional domain or motif other than the enzymatic one, prediction of such additional functions is almost impossible. Identification of protein interaction partners thus provides valuable clues to identify new functions for such a protein (Rao et al., 2014). The interaction of eukaryotic GAPDH with various proteins has been studied for many decades (Kumagai and Sakai, 1983; Glaser et al., 2002; Andrade et al., 2004; Azam et al., 2008; Qvit et al., 2016). On the other hand, similar knowledge on bacterial GAPDH is still very limited. To date, only a single reported study has focused on the identification of intracellular protein interactors of GAPDH derived from *E. coli*. Using immunopurification followed by MS, the authors of that study identified several potential GAPDH interactions, some of which indicated its involvement in DNA repair processes (Ferreira et al., 2013).

Our previously published results clearly indicate that the GAPDH homolog in pathogenic *F. tularensis* – GapA – might fulfill functions other than the enzymatic one. To gain deeper insights into the new intracellular functions of GapA, we performed a quantitative proteomic screen for the potential intracellular interaction partners of *F. tularensis* GapA. We took advantage of SILAC technology and designed an experimental workflow based on the affinity purification of tagged protein bait from the SILAC-labeled bacterial cultures followed by LC-MS/MS analysis. Such approach allows for eliminating the non-specific background that presents the major challenge to traditional AP-MS (Gingras et al., 2007). In this way, we were able to identify several bacterial proteins that seem to bind to the *F. tularensis* GapA. Interestingly, none of the proteins we found here had been identified in the similar study performed with *E. coli* GAPDH (Ferreira et al., 2013). This might be due to the different experimental approach and/or to differences in cellular processes across the various bacterial species. The interaction with six selected proteins was successfully validated by solid-phase ligand binding assay or bacterial two-hybrid assay. To further corroborate our findings, we additionally analyzed changes in the *F. tularensis* proteome induced by the *gapA* gene deletion.

A number of proteins identified in both our proteomic analyses are related to various metabolic processes. This finding is not surprising inasmuch as GAPDH is at the intersection of multiple metabolic pathways and its interaction with other enzymes may point to its regulatory role in these processes. In this context, the levels of some metabolic enzymes changed significantly in response to *gapA* deletion. The identification of proteins involved in amino acid metabolism points to the dispensability of glycolysis for *F. tularensis* demonstrated by several published studies, including ours (Raghunathan et al., 2010; Brissac et al., 2015; Pavkova et al., 2017; Radlinski et al., 2018). *Francisella* is able to utilize amino acids for energy production, especially during its intracellular lifestyle. As the main goal of our study was to obtain information about the GapA functions not related to metabolism, we paid much more attention to those proteins with no direct impact on metabolism.

The cysteine of GAPDH’s catalytic site is very sensitive to overoxidation by reactive H_2_O_2_, resulting in irreversible inactivation of its enzymatic activity. The active site is efficiently protected by *S*-thiolation mediated by glutathione in eukaryotes, Gram-negative and some Gram-positive bacteria, by bacillithiols in most Gram-positives (Kosova et al., 2017) or by mycothiols in *Actinomycetes* (Newton et al., 2008). This modification results in reversible decrease of catalytic activity, and reduction by glutaredoxins or thioredoxins is required for the regeneration of protein function. The *S*-thiolation protects GAPDH also against hypochlorite or sulfide stress (Imber et al., 2016; Peng et al., 2018). Here we demonstrate that thioredoxin is able to form a complex with *F. tularensis* GapA, thus being responsible for maintaining the reduced active protein form. The interaction of thioredoxin with GapA has also been reported in *E. coli* by an interactome analysis *in vivo* (Arts et al., 2016). We further observed that the Δ*gapA* mutant strain is more susceptible to H_2_O_2_ compared to the wild-type strain. GapA seems thus to contribute to cell protection against oxidative stress induced by H_2_O_2_. *F. tularensis* has at its disposal several functional antioxidative systems, and the fully virulent strains of type A and B are highly resistant against the destructive effect of H_2_O_2_ (Lindgren et al., 2007). The mammalian GAPDH is known to act as a redox sensor. The oxidative modification by hydrogen peroxide induces alterations in GAPDH oligomerization that result in its interaction with other proteins and/or translocation into other cellular compartments to fulfill its moonlighting activities. Whether the same is true for bacterial GAPDH homologs remains thus far unknown (Hancock et al., 2006; Russell et al., 2020). The enhanced deleterious effect of H_2_O_2_ on the Δ*gapA* viability may also be related to the decreased levels of OxyR, a transcriptional regulator of genes encoding primary antioxidant proteins, like glutathione, catalase, peroxidase, and others. In *F. tularensis* it regulates the oxidative stress response and provides resistance against reactive oxygen species (Binesse et al., 2015; Ma et al., 2016).

In bacteria, the hydrogen peroxide is able to induce oxidative lesions in the DNA molecule leading to cell death. The damaged DNA is repaired by several DNA repair mechanisms. The potential role of mammalian GAPDH in DNA repair was further indicated in several studies summarized by Kosova et al. (2017), and Ferreira et al. (2013, 2015) described this also for bacterial GAPDH. In *E. coli*, the GAPDH was shown to interact with proteins implicated in SOS response, the single-stranded DNA binding protein, as well as uracil-DNA-glycosylase and endonuclease IV from the base excision repair pathway. Moreover, the *gapA*-silenced *E. coli* cells were more susceptible compared to the wild-type cells to alkylating agents like methyl methanesulfonate but not to H_2_O_2_. In contrast, we were able to identify the UvrA protein from the NER pathway as a putative interaction partner of *F. tularensis* GapA. An absence of GapA, however, had no effect on *F. tularensis* susceptibility to UV irradiation or the alkylating agent methyl methanesulfonate that is the causative agent of DNA lesions repaired by base excision repair (data not shown). Notably, the GapA depletion affected the abundance of proteins of other reparation pathways inclusive of the aforementioned uracil-DNA. Taken together, we observe that GapA seemingly participates in DNA reparation processes in *F. tularensis*. Its involvement is not essential, however, and differs from that in *E. coli*.

The most potent GapA interaction partner detected in our protein interaction studies was the PepSY protein. The presented PepSY domain is widespread across all bacterial species. It occurs mainly in the N-terminal propeptide of the M4 peptidase family, where it seems to inhibit protease activity. Furthermore, this domain is present in a diverse family of secreted and cell wall-associated proteins, where its role has not yet been established. These proteins are hypothesized to contribute to the regulation of protease activity in the cell’s local environment, thus being important for pathogenesis and formation of microbial communities (Yeats et al., 2004). Because proteins with this domain are widely found in both pathogenic and non-pathogenic bacteria, they are not presumed to act as virulence factors. Accordingly, the PepSY-containing protein is not essential for virulence in the *F. tularensis* pathogenic strain. We further undertook to elucidate the role of its interaction with GapA. Because the role of PepSY in GapA secretion was not established in our experiments, we wondered whether, on the contrary, the GapA could assist in PepSY secretion. Similarly, the GAPDH homolog of *Streptococcus pyogenes* has been found to facilitate the export of *Streptococcal pyogenic* exotoxin B, a virulence factor with cysteine protease activity (Jin et al., 2011). Unexpectedly, the results of our comparative proteomics indicated that PepSY seems to be secreted only in the strain not expressing GapA. Thus it might be assumed that the GapA retains this protein inside the cell. The functional importance of this observation remains to be further examined.

This is the first study demonstrating the ability of *F. tularensis* GapA to interact with several bacterial proteins. The identified non-metabolic proteins point to several processes wherein the GapA might assist as a moonlighting protein. Such information is crucial for follow-up studies aimed at identifying further non-enzymatic functions of *F. tularensis* GapA at the intracellular level.

## Data Availability Statement

The datasets presented in this study can be found in online repositories. The names of the repository/repositories and accession number(s) can be found at: https://www.ebi.ac.uk/pride/archive/, PXD019739.

## Ethics Statement

The animal study was reviewed and approved by Ethical Committee of the Faculty of Military Health Sciences, University of Defence, Czechia.

## Author Contributions

MK and IP designed and performed the research, analyzed the data, and wrote the manuscript. ML and PR performed the research and analyzed the data. JS reviewed and edited the manuscript. All authors contributed to the article and approved the submitted version.

## Conflict of Interest

The authors declare that the research was conducted in the absence of any commercial or financial relationships that could be construed as a potential conflict of interest.

## Footnotes

1 https://www.evotec.com/en/execute/science-pool/resources

2 https://www.uniprot.org/uniprot/A0A0B3VXZ9
