# Supplementary material for: Identification of Bacterial Protein Interaction Partners Points to New Intracellular Functions of Francisella tularensis Glyceraldehyde-3-Phosphate Dehydrogenase
Source: Front Microbiol. 2020 Sep 10;11:576618. doi: 10.3389/fmicb.2020.576618 (PMC7513575; doi:10.3389/fmicb.2020.576618)
Supplement: Supplementary file 1 [file Table_1.DOCX]

***Supplementary Material***

1. **Supplementary Tables**

**Supplementary Table 1** The F. tularensis and E. coli strains and plasmids used in this study

| ***Francisella tularensis* strain** | *Genotype or description* | *Reference* |
| --- | --- | --- |
| FSC200 | *F. tularensis subsp. holarctica;*  *clinical isolate* | *Francisella* strain  Collection (Johansson et al., 2000) |
| *∆gapA* (deletion mutant) | *∆FTS_1117/FSC200* | (Pavkova et al., 2017) |
| *∆pepSY* (deletion mutant) | *∆FTS_1731/FSC200* | This study |
| GapA+StrepTag | *GapA/S* | This study |
| ***E.coli* strain** |  |  |
| K12 (C2984H) | *F' proA+B+ lacIq ∆lacZM15 / fhuA2 ∆(lac-proAB) glnV galK16 galE15 R(zgb-210::Tn10)TetS endA1 thi-1 ∆(hsdS-mcrB)5* | New England BioLabs |
| S17 - 1λ pir | *recA, thi, pro, hsdR−M+,*  *<RP4:2-Tc:Mu:Km:Tn7>TpR,*  *SmR* | (Simon et al., 1983) |
| TOP10 | *F− mcrA*  *1(mrr-hsdRMS-mcrBC),*  *φ80lacZ1M15 1lacX74 recA1*  *deoR araD139 (1ara-leu)7697*  *galU galK rpsL (Smr ) endA1*  *nupG* | Invitrogen |
| NiCo21 | can::CBD fhuA2 [lon] ompT gal (λ DE3) [dcm] arnA::CBD slyD::CBD glmS6Ala ∆hsdS λ DE3 = λ sBamHIo ∆EcoRI-B int::(lacI::PlacUV5::T7 gene1) i21 ∆nin5 | New England BioLabs |
| BTH101 | F-, *cya-99*, *araD139, galE15, galK16, rpsL1 (Str r)*, *hsdR2, mcrA1, mcrB1* | Euromedex |
| ***Plasmid*** |  |  |
| pET-28b | *Plasmid for protein over expression* | Addgene |
| pCR®4.0-TOPO | *TOPO-cloning vector. AmpR,*  *KmR* | Invitrogen, Carlsbad, CA, USA |
| pDM4 | *Suicide plasmid. sacB; mobRP4;*  *oriR6K; CmR* | (Milton et al., 1996) |
| pKT25 | *BACTH vectors* | Euromedex |
| pUT18C | *BACTH vectors* | Euromedex |

Johansson, A., Berglund, L., Eriksson, U., Göransson, I., Wollin, R., Forsman, M., et al. (2000). Comparative Analysis of PCR versus Culture for Diagnosis of Ulceroglandular Tularemia. *J Clin Microbiol* 38, 22–26.

Milton, D. L., O’Toole, R., Horstedt, P., and Wolf-Watz, H. (1996). Flagellin A is essential for the virulence of Vibrio anguillarum. *J Bacteriol* 178, 1310–1319.

Pavkova, I., Kopeckova, M., Klimentova, J., Schmidt, M., Sheshko, V., Sobol, M., et al. (2017). The Multiple Localized Glyceraldehyde-3-Phosphate Dehydrogenase Contributes to the Attenuation of the Francisella tularensis dsbA Deletion Mutant. *Front Cell Infect Microbiol* 7, 503. doi:10.3389/fcimb.2017.00503.

Simon, R., Priefer, U., and Pühler, A. (1983). A Broad Host Range Mobilization System for In Vivo Genetic Engineering: Transposon Mutagenesis in Gram Negative Bacteria. *Nat Biotechnol* 1, 784–791. doi:10.1038/nbt1183-784.

**Supplementary Table 2** Primers used in this study.

| *Protein name* | *FTS locus tag* | *Tag* | *Primer designation* | *5´- 3´ Sequence* |
| --- | --- | --- | --- | --- |
| Glyceraldehyde-3-phosphate dehydrogenase (GapA) | FTS_1117 | Strep-Tag | A | TCATTTATATCACCTATAATGTTTAATGACTCCAAGCACTTAGAGCTCCGAAGTACTCTACTAC |
|  |  |  | B | GTAGTAGAGTACTTCGGAGCTCTAAGTGCTTGGAGTCATTAAACATTATAGGTGATATAAATGA |
|  |  |  | C | TCATTTATATCACCTATAATGTTTATTTTTCAAATTGAGGATGACTCCAAGCACTTAGAGCTCC |
|  |  |  | D | GGAGCTCTAAGTGCTTGGAGTCATCCTCAATTTGAAAAATAAACATTATAGGTGATATAAATGA |
|  |  |  | Rev_XhoI | *CTCGAG*TATATAGCTTCGCAATTGA  GTAA |
|  |  |  | For_SpeI | *ACTAGT*ATCTCATCCGCAACAAC  ATAG |
|  |  | *BACTH -* pKT25, pUT18C | Rev_Gths | GCG*GGATCC*TAGAGTTGCAATTAATGGTTTCGGTAGAAT |
|  |  |  | For_Gths | GCG*GGTACC*CATAGAGCTCCGAAGTACTCTACTACTCTAA |
| UvrABC system protein A (UvrA) | FTS_1439 | *BACTH -* pKT25, pUT18C | Rev_Uths | GCG*GGTACC*TGGAGTAAATCTTTAAGATACTTACCA |
|  |  |  | For_Uths | CGC*GGATCC*TAAAAAAATTATCGTCAAGGGCGC |
| Transcription termination factor Rho (Rho) | FTS_0609 | His-tag | For_rho | GCG*CCATGG*TGAACTTAAATGAATTAAAGTATAAATC |
|  |  |  | Rev_rho | CGC*CTCGAG*AATAGCGCCTCTTTTCATAGTTTC |
| Thioredoxin | FTS_0610 | His-tag | Rev_ trxA | CGC*CTCGAG*TAGATATTTGTCTACGATTGAGATT |
|  |  |  | For_ trxA | CGC*AAGCTT*ATGTCAAAATGTATCGATATATCAG |
| YCII-related domain protein | FTS_1107 | His-tag | For_Unch | CGC*CCATGG*TGCAAATTCATATAATTGATATTCACTA |
|  |  |  | Rev_Unch | GCG*CTCGAG*TGAGTTAGCAAGTTCTGGGATA |
| Cytosol aminopeptidase | FTS_1077 | His-tag | For_CAP | CGC*CCATGG*TGTATATATCAACCAAATTAGAGTGTTT |
|  |  |  | Rev_CAP | GCG*CTCGAG*AGCTGGAAACTTATCCTTAAGC |
| Hypothetical protein (PepSY) | FTS_1731 | His-tag | For_ pepSY | CGC*CCATGG*TGATTATGTTTTGGAGGTTATATATGA |
|  |  |  | Rev_ pepSY | CGC*CTCGAG*ATCATCATAACTTATCTGTGAAATT |
|  |  | *gene deletion* | A | GCATGT*CTCGAG*CATATACTGCTGTCAAGAAT |
|  |  |  | B | ATCATCATAACTCTTATTCATATATAACCTCCAAAA |
|  |  |  | C | TATATATGAATAAGAGTTATGATGATTAGCTAAAAATA |
|  |  |  | D | GCATGT*GAGCTC*GCGGCACTCCTTCTCTGTTT |

Note: Restriction sites for selected endonucleases on primers A and D are in italic; complementary parts of primer B and C are underlined.
